# Can high private cough syrup sales act as a proxy for missed TB notifications in TB surveillance?

**DOI:** 10.5588/pha.25.0033

**Published:** 2025-12-03

**Authors:** K. Gupta, H.D. Shewade, P. Manickam, P. Soni, V.N. Baig, R. Dagar, M. Parmar, Y. Saxena, S. Charan, H. Abdullah, A.K. Bhardwaj

**Affiliations:** 1 Department of Community Medicine, Geetanjali Medical College and Hospital, Rajasthan, India;; 2 Division of Health Systems Research, ICMRNational Institute of Epidemiology (ICMR-NIE), Chennai, India;; 3 State TB Cell, Directorate of Medical & Health Services, Jaipur, India;; 4 Department of Community Medicine, Rajasthan University of Health Sciences College of Medical Sciences, Jaipur, India;; 5 Department of Community Medicine, Ananta Institute of Medical Sciences and Research Centre, Rajsamand, India;; 6 Department of Community Medicine, GMERS Medical college, Godhra, India;; 7 Khushi Baby, Jaipur, India;; 8 Department of Community Medicine, M.M. Medical college, Solan, India.

**Keywords:** tuberculosis, SORT IT, antitussives, TB burden, Rajasthan, India

## Abstract

**BACKGROUND:**

Rajasthan in India, is a high TB burden state with a high prevalence to notification ratio. This gap calls for alternative strategies to find the non-notified people with TB. Because cough is the most prominent symptom of TB and cough syrups are one of the highest over the counter drugs in India, its sale might be used as a proxy for missed TB cases.

**METHODS:**

This was an ecological study to assess the correlation between cough syrup sales and missed TB notifications in the private sector. We calculated the missed TB notification rate as the difference between the estimated people on TB treatment and TB notification in the private sector from January 2021 to March 2023, across all districts of Rajasthan (n = 33). We analysed district level mean quarterly cough syrup sales and TB notification rates/100,000 population.

**RESULTS:**

We found positive correlation between cough syrup sales and TB notification [overall (r = 0.43), private (r = 0.63) and missed private TB notification (r = 0.39)]. Based on this analysis, missed TB notification rates were 7x more than the reported notifications in the private sector.

**CONCLUSION:**

We recommend a private sector-based TB surveillance system with TB screening for people who approach pharmacies for cough syrup.

Globally, the estimated annual TB incidence rate is 134/100,000 and 2.4 million are missed annually by the national TB programs.^1^ India constitutes 26% of the global TB burden, with an estimated annual incidence of TB of 195/100,000 (0.3 million are missed).^1^At least half of the people with TB symptoms in India seek care from the private sector.^2,3^ Although the public sector has traditionally been at the centre of the TB response, it is the private sector that often serves as the first point of contact for individuals seeking healthcare, particularly in high TB burden countries where the public sector may be under-resourced, stigmatizing or difficult to access.^4^

India’s national strategic plan for TB elimination (2017–2025) established strategies to ensure that patients reaching the private sector receive timely and quality-assured services. This includes diagnosis and treatment, protection from high out-of-pocket expenditure, management of comorbidities, contact investigation, TB prevention therapy, counselling, adherence support and monitoring, nutritional support, and outcome reporting.^5^ Despite the efforts of India’s national TB elimination program (NTEP), private sector engagement remains suboptimal. Over half of the total TB notifications in India are accounted by 5 states and Rajasthan is one of them.^6^ Rajasthan is the largest state (by area, arid to semi-arid) in India (north-west) with 80 million population and ≈165,000 annual TB notifications.^7^ While the estimated prevalence of TB (2019–2021) in Rajasthan is 484/100,000, the state notifies 204/100,000 annually (51/100,000 by the private sector).^7^ This indicates a high prevalence to notification ratio (2.3:1) when compared to the national estimate (1.8:1). This gap requires us to assess alternative strategies to augment NTEP initiatives.^8^

Many infectious diseases have benefitted from the use of alternate strategies, such as surveillance for fever with rash for measles and acute flaccid paralysis for poliomyelitis, or use of drug sales at pharmacies to trace cases of diabetes, asthma, drug abuse, depression and cardio vascular diseases.^9^ In the context of ending the TB epidemic, such an approach could make use of common symptoms (e.g., cough, fever, night sweats and weight loss) and help-seeking behaviour.^10^ Of these, cough is the key symptom of pulmonary TB (PTB) and is associated with transmission.^11^ Modern medicine cough syrups are the second most common drug used for self-medication by the public.^10,12^ Hence, sales data of cough syrups might serve as a proxy indicator for high TB notification to identifying missed TB cases. Earlier screening of patients with cough will help diagnose PTB sooner.^13^ However, there are limited studies on whether cough syrup sales data at the population level may be used as a proxy to either guide or strengthen private sector engagement to improve TB notification.

We therefore set out to explore the relationship between cough syrup sales and TB notification in the private sector in Rajasthan. In this statewide study, the specific objectives were to (1) describe the district level mean quarterly private cough syrup sales, and overall, private and missed private TB notification rates and (2) estimate the correlation between district level mean quarterly private cough syrup sales and overall, private and missed private TB notification rate (all expressed as per 100,000 population).

## METHODS

### Study setting

This was an ecological study for the period January 2021 to March 2024 (13 quarters). The quarterly district level aggregate data was the unit of analysis. The study utilized data from all the 33 districts of Rajasthan as of 2021–22 (see Box 1). In March 2023, with the announcement of the creation of new districts, the number of districts increased from 33 to 50 (see Box 1).^14,15^ However, for our study, we retained the previous 33 district level units across all the 13 quarters.

BOX 1. Districts in Rajasthan, India.^14,15^

**Districts of Rajasthan as of 2021 to 2022 (N=33)**

Ajmer, Alwar, Banswara, Baran, Barmer, Bharatpur, Bhilwara, Bikaner, Bundi, Chittorgarh, Churu, Dausa, Dholpur, Dungarpur, Hanumangarh, Jaipur, Jaisalmer, Jalore, Jhalawar, Jhunjhunu, Jodhpur, Karauli, Kota, Nagaur, Pali, Pratapgarh, Rajsamand, SawaiMadhopur, Sikar, Sirohi, Sri Ganganagar, Tonk and Udaipur.

**17 newdistricts in Rajasthan after March 2023**

Anupgarh, Balotra, Beawer, Kekri, Jaipur rural, Dudu, Kotputli-Behror, Neem kathana, Khairtal-Tijara, Sanchore, Didwana-kuchaman, Shahpura, Jodhpur rural, Phalodi, Salumber, Gangapur, Deeg

At the country level, the NTEP is led by the central TB division under the Ministry of Health and Family Welfare. The Rajasthan state TB cell and the district TB cells govern the activities of the program at the state and district level, respectively. At the sub-district/block level, activities are organized under the TBunit.^5^ The state TB cell implements a patient provider incentive scheme where they engage private health care providers directly (without third-party agency/non-governmental organization as an intermediary as in patient provider support agency scheme) and incentivize TB notification, TB services and outcome declaration. Food safety and drug control Commissionerate is the nodal regulatory agency under Medical and Health Department of Government of Rajasthan for regulating the manufacture of drugs, medical device and cosmetics and sale of drugs.

### Private sector cough syrup sales data

Cough syrup contains muco-active agents and is taken to stop coughing,^16^ and sales by privately owned pharmacies or chemist shops are termed ‘private cough syrups’. We collected district level cough syrups sales data from the private sector from drug controller under food safety and drug control Commissionerate, Government of Rajasthan. District wise monthly data of various brands of cough syrups were shared via email to the principal investigator. These monthly cough syrup sales were converted into litres per quarter per district per 100,000 population. District population data used in calculation was the average of the three years, 2021–2023.^17^

### Private sector TB notification rate

Monthly district level private TB notification numbers were extracted from *Ni-kshay* portal. These numbers were converted into district level quarterly private TB notification rate per 100,000 population.

### Estimated people on TB treatment in private sector

This was calculated based on an indirect method of calculating TB incidence.^18^ This involved obtaining monthly district level, private sector, sales data on rifampicin containing anti-TB drugs. We used this sales data to compute the numbers of rifampicin drug packs sold per quarter. This value was then used to calculate estimated people on TB treatment in the private sector per 100,000 population.

### Missed TB notification rate in private sector

We calculated the missed private TB notification rate per 100,000 population (quarterly and district wise) as the difference between estimated people on TB treatment and actual notifications in private sector.

### Statistical analysis

IBM SPSS software version 23 was used for analysis. At district level, the quarterly mean cough syrup sales in the private sector and TB notification rate (overall, private and missed private) were described as trends over 13 quarters. The same was described at district level for all 13 quarters combined (mean) using *Datawrapper* choropleth maps (https://www.datawrapper.de/maps/choropleth-map). We calculated Pearson correlation coefficient (r) between district level quarterly mean cough syrup sales in private sector and TB notification rates (overall, private and missed private). All quarterly indicators were non-annualized and per 100,000 population.

### Ethical statement

The study was approved by the Institutional Human Ethics Committee of ICMR-National Institute of Epidemiology, Chennai, India (NIE/IHEC/A/202408-04, dated 11 September 2024). As the study involved the use and analysis of aggregate data from existing secondary data sources, we sought and obtained waiver for informed consent.

## RESULTS

The district level mean quarterly private cough syrup sales was 89 litres/100,000 population and it ranged from 64 litres (lowest) in April–June 2023 to 125 litres (highest) in January–March 2023. District level mean quarterly overall notification rate was 43/100,000 population: lowest in April–June 2021 and highest in April–June 2022 and it was 12/100,000 population in the private sector: lowest in January–March 2023 and highest in July–September 2021. District level mean quarterly missed private notification rate was 81/100,000 population: lowest in January–March 2023 and highest in January–March 2022 (https://figshare.com/s/9191f07bf0bcbe495055). None of the trend lines followed any specific pattern. All four variables have mostly peaked in the 2^nd^ quarter of the years depicted.

District wise mean quarterly cough syrup sales reveals Jaipur and Alwar had the highest private cough syrup sales and highest overall and private notifications (https://figshare.com/s/2eb03d6cbf92298acc18). Other districts, such as Jaipur, Bikaner, Ajmer, Kota, Udaipur, Karauli and Sawai Madhopur also had high private cough syrup sales as well as high overall, private and missed private notifications. Nagaur, Chittorgarh and Pratapgarh had the lowest private cough syrup sales as well as lowest overall, private and missed private notifications.

### Correlation between private cough syrup sales and TB notification rates

**FIGURE 1.**
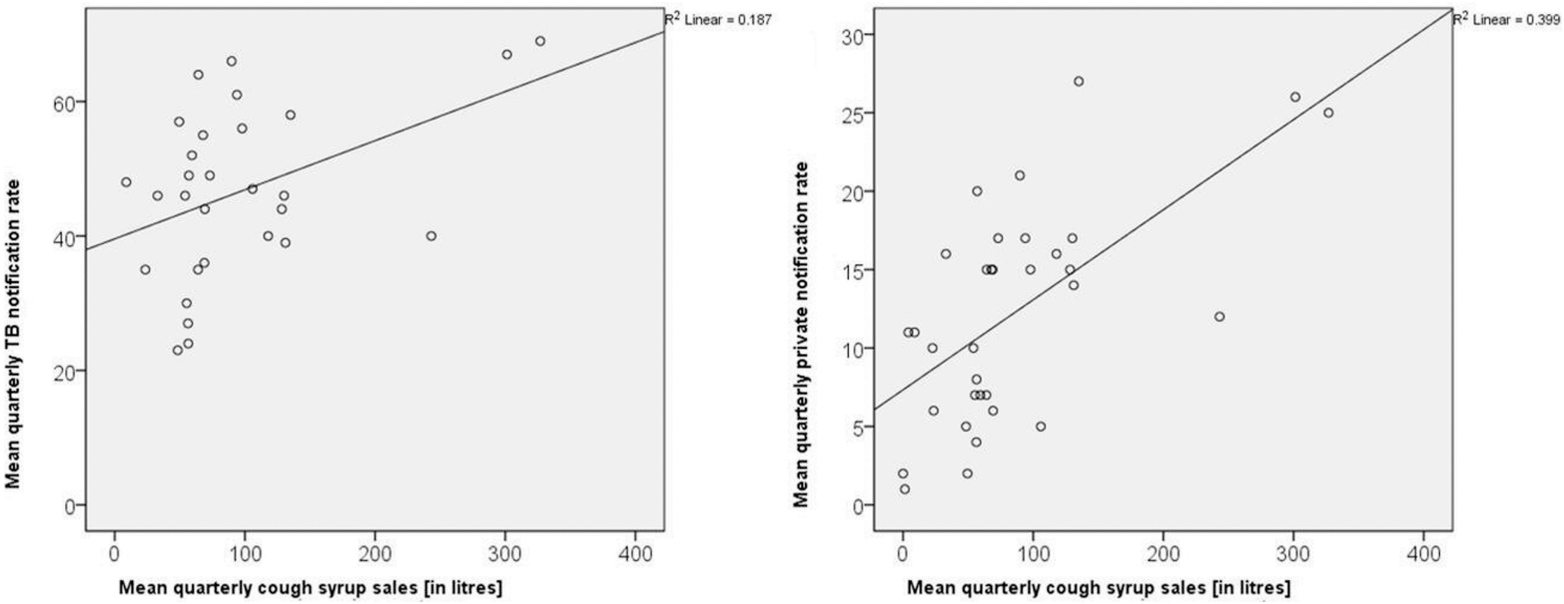
Correlation between district level cough syrup sales from private sector (mean in litres/100,000 population, quarterly^**A**^) and TB notification rates (total notification, private sector notification/100,000 population, quarterly^**A**^), across 32 districts of Rajasthan, India^**B**^ from January 2021 to March 2024. ^**A**^Non-annualized; ^**B**^data of cough syrup sales was not available for Jaisalmer; for overall TB notification rate, r = 0.43; p = 019; for private TB notification rate, r = 0.61; p = <0.001

**FIGURE 2.**
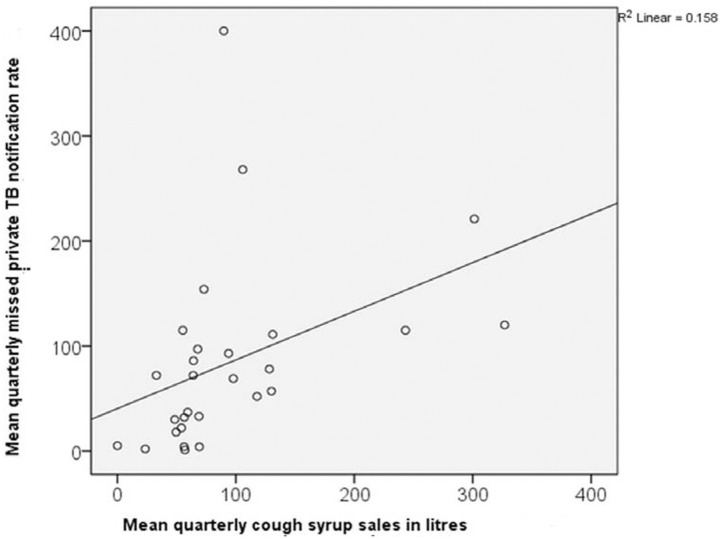
Correlation between district level cough syrup sales from the private sector (mean in litres/100,000 population, quarterly^**A**^) and missed TB notification rates/100,000 population quarterly^**A**^ across 28 districts of Rajasthan, India^**B**^ from January 2021 to March 2024. ^**A**^Non-annualized; r = 0.39, p = 0.036; ^**B**^data of cough syrup sales was not available for Jaisalmer; data of private rifampicin containing anti-TB drug sales was not available for Baran, Bundi, Jaisalmer and Rajsamand (Alwar was an outlier and hence, removed).

There was a positive correlation between district level mean quarterly private cough syrup sales and mean quarterly overall (r = 0.43, p = 0.019), private (r = 0.61, p = <0.001) and missed private notification rate (r = 0.39, p = 0.036) – see Figure 1 and Figure 2. This positive correlation corroborates the findings of descriptive analysis above.

## DISCUSSION

This was the first state-wide study conducted over more than three years in the context of high overall missed TB and the low contribution of private sector TB notification. Our hypothesis was that people living with TB and having a cough are self-medicating with cough syrups instead of being tested for TB. At the district level in the private sector, the missed TB notification was found to be 7x more than the reported private sector notification. Many people with TB are being managed in the private sector but not being notified. The higher the cough syrup sales in a district, the higher the TB notifications (overall and private) and missed private TB notifications.

Cough syrup sales and TB notification data were adjusted for the district size by calculating the indicators per 100,000 population. However, there were some limitations. First, as with any operational research using routine data, there is potential for errors in the data collected. Second, being an ecological study, ecological fallacy cannot be ruled out. The population level inferences may not apply at individual level. Other district level confounders, like healthcare access, population health-seeking behaviour, or socio-economic status were not adjusted for due to lack of structured data. Third, we were unable to obtain cough syrup sales data under the traditional Indian medical systems termed *Ayush* (Ayurveda, Yoga, Unani, Siddha and Homeopathy), operating in the public sector. Therefore, the reported cough syrup sales data in our study is an underestimate. Finally, there are limitations to the indirect method to estimate people on TB treatment in the private sector.^18^ Not all rifampicin containing drug sales data is reflective of a person with TB as some is used in certain skin diseases. Also, empirical pharmacological treatment for TB is started for many and then stopped after a few months when there is no clinical improvement (possible diagnosis other than TB). Although this is adjusted for in the calculation, it could still be a limitation,^18^ but it is unlikely to be different across districts. Limitations notwithstanding, the findings have policy and practice relevance as discussed below.

### Policy and practice relevance

First, based on drug-sales data, in Rajasthan, many people with TB are being managed in the private sector but not being notified. In India, around 80% of TB patients seek medical advice from a private practitioner before approaching the public sector (because of accessibility, availability, confidentiality and personalized care) and among these, 40% have private TB treatment.^19^ Despite this, the barriers to private sector engagement in notifying people with TB remain and have been extensively studied in India.^20–26^ Lack of training in using the *Ni-kshay* portal for notifications, high patient load, scarcity of time to report, unmotivated practitioners and fear of losing patients are just some of the reasons for under reporting from the private sector.^20,21,23,26,27^ This suggests the need to review the existing incentive scheme for private sector engagement in Rajasthan. Additionally, TB surveillance at the pharmacy level should be considered to identify people with TB who are taking treatment in the private sector but are not notified.^28^ Himachal Pradesh, a hilly state in north India, has started to implement this as per their ‘TB free Himachal campaign’.^29^ With the involvement of the state drug control department, pharmacists can share data on the people who are consuming cough syrups and these people can be tracked and see if they fall into the category of presumptive PTB cases. Tracking of the cough syrup buyers can be done by health care workers. Strict laws can ensure the privacy of the people tracked under this mechanism.

Second, districts with high cough syrup sales in the private sector showed high overall TB notification. Because cough is the most common symptom among people with TB, they first tend to self-medicate and then seek medical care after a delay.^30^ A study from Shenzhen, China, reported that the use of over the counter cough syrups is associated with pre-diagnosis delay.^31^ Findings from a meta-analysis revealed that 42% of PTB patients delayed seeking care by 1 month or more.^32^ Hence, to reduce pre-diagnosis delay, it has been suggested that people who seek cough syrup at pharmacies in high TB prevalence countries like India should be screened for TB regardless of cough duration.^13^ An intervention study in the city of Patna in India, involving pharmacies for TB screening and referral found an increase of TB notification by 62x compared to a control group.^33^ Himachal Pradesh has mandated the provision of mobile numbers of people seeking cough syrups from pharmacies. The district TB cell conducts outreach activities involving sputum collection and transport for TB diagnosis.

Third, the higher the level of cough syrup sales, the higher the missed private TB notifications. Based on this finding, we recommend the inclusion of quarterly cough syrup sales per 100,000 population at district level as part of routine surveillance within state NTEPs. In resource constrained settings, districts within a state with higher cough syrup sales may be prioritized for initiatives to improve private sector engagement, TB surveillance through pharmacies and TB screening for people. Such initiatives may be guided by implementation and operational research. During 2021–2023, the performance of Rajasthan in notifying people with TB from private sector (51–57/100,000) was similar to the national level performance (49–59/100,000).^7,34,35^ However, based on our findings, there is scope to improve and our suggestions may beapplicable to other states and countries with high TB burden and missed notifications from the private sector.

## CONCLUSION

In Rajasthan, India, based on rifampicin containing anti-TB drug sales, missed TB notifications was 7x the reported private TB notification rate. At district level, we identified a positive correlation between cough syrup sales and overall notification and missed TB notifications in the private sector. District level cough syrup sales per 100,000 in the private sector may be included as part of routine TB surveillance. In resource constrained settings, initiatives to engage private physicians and pharmacies should be prioritized in districts with high cough syrup sales.
